# 1,2:3,5-Bis[(4-*tert*-butyl­phen­yl)boranedi­yl]-α-d-glucofuran­ose

**DOI:** 10.1107/S1600536810046222

**Published:** 2010-11-13

**Authors:** Marek Dąbrowski, Sergiusz Luliński, Janusz Serwatowski, Agnieszka Wilmowicz

**Affiliations:** a Physical Chemistry Department, Faculty of Chemistry, Warsaw University of Technology, Noakowskiego 3, 00-664 Warsaw, Poland

## Abstract

The crystal structure of the title compound, C_26_H_34_B_2_O_6_, comprises two crystallographically independent mol­ecules. In the crystal, the mol­ecules are linked by multiple inter­molecular O—H⋯O and C—H⋯O hydrogen bonds into a two-dimensional array.

## Related literature

For the structural characterization of related monosaccharide boronates, see: Chandran & Nangia (2006) ▶; Draffin *et al.* (2004 ▶). For complexes of boronic acids with glucose, see: Hall (2005 ▶).
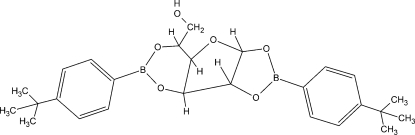


## Experimental

### 

#### Crystal data

C_26_H_34_B_2_O_6_
*M* *_r_* = 464.15
Monoclinic, 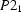

*a* = 11.2372 (3) Å
*b* = 10.1910 (3) Å
*c* = 22.5084 (7) Å
β = 104.103 (3)°
*V* = 2499.93 (13) Å^3^
*Z* = 4
Mo *K*α radiation
μ = 0.09 mm^−1^
*T* = 100 K
0.60 × 0.30 × 0.22 mm

#### Data collection

Kuma KM-4-CCD diffractometer
Absorption correction: multi-scan (*CrysAlis RED*; Oxford Diffraction 2005 ▶) *T*_min_ = 0.95, *T*_max_ = 0.98
47322 measured reflections
6519 independent reflections
5409 reflections with *I* > 2σ(*I*)
*R*_int_ = 0.025

#### Refinement

*R*[*F* ^2^ > 2σ(*F* ^2^)] = 0.031
*wR*(*F* ^2^) = 0.072
*S* = 0.99
6519 reflections
627 parameters
1 restraint
H-atom parameters constrained
Δρ_max_ = 0.24 e Å^−3^
Δρ_min_ = −0.19 e Å^−3^

### 

Data collection: *CrysAlis CCD* (Oxford Diffraction, 2005 ▶); cell refinement: *CrysAlis RED* (Oxford Diffraction, 2005 ▶); data reduction: *CrysAlis RED*; program(s) used to solve structure: *SHELXS97* (Sheldrick, 2008 ▶); program(s) used to refine structure: *SHELXL97* (Sheldrick, 2008 ▶); molecular graphics: *DIAMOND* (Brandenburg, 1999 ▶); software used to prepare material for publication: *SHELXL97*.

## Supplementary Material

Crystal structure: contains datablocks I, global. DOI: 10.1107/S1600536810046222/pb2045sup1.cif

Structure factors: contains datablocks I. DOI: 10.1107/S1600536810046222/pb2045Isup2.hkl

Additional supplementary materials:  crystallographic information; 3D view; checkCIF report

## Figures and Tables

**Table 1:** Hydrogen-bond geometry (Å, °)

*D*—H⋯*A*	*D*—H	H⋯*A*	*D*⋯*A*	*D*—H⋯*A*
O6*A*—H6*A*⋯O6*B*	0.84	2.07	2.8660 (17)	158
O6*B*—H6*B*⋯O1*A*^i^	0.84	2.47	3.1131 (17)	134
C1*B*—H1*B*⋯O6*A*	1.00	2.37	3.318 (2)	159
C2*B*—H2*B*⋯O4*B*^ii^	1.00	2.41	3.379 (2)	162
C4*A*—H4*A*⋯O5*B*^iii^	1.00	2.44	3.246 (2)	137
C4*B*—H4*B*⋯O6*A*^iv^	1.00	2.51	3.231 (2)	129
C3*A*—H3*A*⋯O1*B*^i^	1.00	2.55	3.480 (2)	155
C6*A*—H6*A*1⋯O4*A*^i^	0.99	2.60	3.499 (2)	150
C1*A*—H1*A*⋯O6*B*	1.00	2.62	3.553 (2)	155
C2*A*—H2*A*⋯O4*A*^i^	1.00	2.64	3.587 (2)	159
C14*A*—H14*A*⋯O2*A*^i^	0.95	2.66	3.433 (2)	139
C3*A*—H3*A*⋯O4*B*^i^	1.00	2.68	3.442 (2)	133
C3*B*—H3*B*⋯O4*A*^v^	1.00	2.72	3.522 (2)	137

Symmetry codes: (i) 
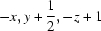
; (ii) 
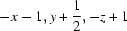
; (iii) 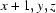; (iv) 
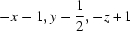
; (v) 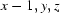.

**Fig. 1.**
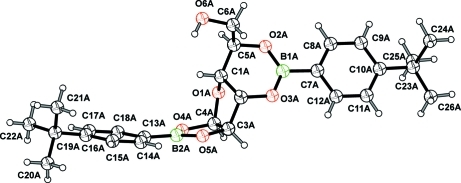
The molecular structure of molecule A of (I) showing the atom-labelling scheme. Displacement ellipsoids for all non-H atoms are drawn at the 50% probability level.

**Fig. 2.**
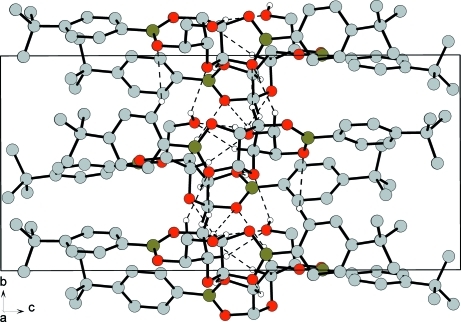
The crystal packing diagram for (I) showing hydrogen-bonding and C–H···O interactions (dashed lines).

## supplementary crystallographic information

### Comment

The interaction of boronic acids with sugars is of great importance due to potential applications in medicine. The work in the area is focused mainly on complexes of boronic acids with glucose (Hall, 2005). Despite a signifcant progress in the field only two crystal structures of 2:1 boronic acid–glucose complexes were reported (Chandran & Nangia, 2006). They involved simple phenylboronic acid and its 4-methyl congener. The molecule of the title compound (I) features the glucose backbone in its furanose form, which resembles the situation observed in crystal structures for analogous complexes. There are only slight differences in the metric parameters of two independent molecules. One of boron atoms is bonded to O4 and O5 atoms, which results in the formation of a 5-membered ring. The second boron atom is bonded to O2 and O3 atoms; as a result a 6-membered ring is formed. Both boron-containing heterocycles are approximately coplanar with the adjacent phenyl rings. The supramolecular arrangement in (I) is dictated by one relatively strong O–H···O hydrogen bond which links the terminal hydroxyl groups of two independent molecules. The weaker O–H···O hydrogen bond is formed between the H6B atom and the ring O1A atom. These two interactions are complemented by the set of multiple C–H···O contacts (Table 1). The shortest one (2.37 Å) is observed between the H1B and the O6A atoms. Unlike the tolyl groups in the related compound (Chandran & Nangia, 2006), the *tert*-butyl groups in (I) are not involved in C–H···O interactions. As a result, a two-dimensional array aligned parallel to the (001) plane is formed by atoms from central glucofuranose cores of both independent molecules whereas a further three-dimensional assembly is achieved only by weak van der Waals interactions of external *tert*-butyl groups.

### Experimental

A mixture of α-D-glucose (3.6 g) and 4-(*tert*-butyl)phenylboronic acid (3.55 g) in 1,4-dioxane (30 ml) was stirred for 6 hrs at 50 *^o^*C. The resulting solution was concentrated. The residue was extracted with benzene (2 *x* 30 ml). Combined extracts were filtered. Evaporation yielded a solid which was washed with pentane (20 ml). Yield of (I) 4.0 g, m.p. 410 K. ^1^H NMR (CDCl_3_): 7.79 (m, 4 H), 7.45 (d, 2 H), 7.40 (d, 2 H), 6.18 (d, 1 H), 5.00 (d, 1 H), 4.70 (d, 1 H), 4.47 (t, 1 H), 4.27 (d. 1H), 3.86 (m, 1 H), 3.73 (m, 1 H), 1.81 (t, 1 H),1.33 (s, 9 H), 1.32 (s, 9 H) p.p.m.; ^11^B NMR: 26.0 p.p.m.. Crystals suitable for single-crystal X-ray diffraction analysis were grown by slow evaporation of a solution of (I) (0.2 g) in benzene (10 ml).

### Refinement

All hydrogen atoms were located geometrically with C—H  =  0.95—1.00 Å and O—H  = 0.84 Å, and were included in the refinement in the riding model approximation with U(H) set to 1.2—1.5U_eq_(C/O).

### Crystal data

**Table:**

C_26_H_34_B_2_O_6_	*D*_x_ = 1.233 Mg m^−^^3^
*M**_r_* = 464.15	Melting point: 480 K
Monoclinic, *P*2_1_	Mo *K*α radiation, λ = 0.71073 Å
*a* = 11.2372 (3) Å	Cell parameters from 28404 reflections
*b* = 10.1910 (3) Å	θ = 2.2–28.8°
*c* = 22.5084 (7) Å	µ = 0.09 mm^−^^1^
β = 104.103 (3)°	*T* = 100 K
*V* = 2499.93 (13) Å^3^	Prismatic, colourless
*Z* = 4	0.60 × 0.30 × 0.22 mm
*F*(000) = 992	

### Data collection

**Table:**

Kuma KM-4-CCD diffractometer	6519 independent reflections
Radiation source: fine-focus sealed tube	5409 reflections with *I* > 2σ(*I*)
graphite	*R*_int_ = 0.025
Detector resolution: 8.6479 pixels mm^-1^	θ_max_ = 28.6°, θ_min_ = 2.7°
ω scans	*h* = −14→15
Absorption correction: multi-scan (*CrysAlis RED*; Oxford Diffraction 2005)	*k* = −13→13
*T*_min_ = 0.95, *T*_max_ = 0.98	*l* = −29→30
47322 measured reflections	

### Refinement

**Table:**

Refinement on *F*^2^	Primary atom site location: structure-invariant direct methods
Least-squares matrix: full	Secondary atom site location: difference Fourier map
*R*[*F*^2^ > 2σ(*F*^2^)] = 0.031	Hydrogen site location: inferred from neighbouring sites
*wR*(*F*^2^) = 0.072	H-atom parameters constrained
*S* = 0.99	*w* = 1/[σ^2^(*F*_o_^2^) + (0.0459*P*)^2^] where *P* = (*F*_o_^2^ + 2*F*_c_^2^)/3
6519 reflections	(Δ/σ)_max_ = 0.001
627 parameters	Δρ_max_ = 0.24 e Å^−^^3^
1 restraint	Δρ_min_ = −0.19 e Å^−^^3^

### Special details

**Table:**

Geometry. All e.s.d.'s (except the e.s.d. in the dihedral angle between two l.s. planes) are estimated using the full covariance matrix. The cell e.s.d.'s are taken into account individually in the estimation of e.s.d.'s in distances, angles and torsion angles; correlations between e.s.d.'s in cell parameters are only used when they are defined by crystal symmetry. An approximate (isotropic) treatment of cell e.s.d.'s is used for estimating e.s.d.'s involving l.s. planes.
Refinement. Refinement of *F*^2^ against ALL reflections. The weighted *R*-factor *wR* and goodness of fit *S* are based on *F*^2^, conventional *R*-factors *R* are based on *F*, with *F* set to zero for negative *F*^2^. The threshold expression of *F*^2^ > σ(*F*^2^) is used only for calculating *R*-factors(gt) *etc*. and is not relevant to the choice of reflections for refinement. *R*-factors based on *F*^2^ are statistically about twice as large as those based on *F*, and *R*- factors based on ALL data will be even larger.

### Fractional atomic coordinates and isotropic or equivalent isotropic displacement parameters (Å^2^)

**Table:**

	*x*	*y*	*z*	*U*_iso_*/*U*_eq_	
B1A	−0.05450 (17)	0.11527 (19)	0.33127 (9)	0.0162 (4)	
B2A	0.15751 (16)	0.0744 (2)	0.57760 (9)	0.0167 (4)	
C1A	−0.05567 (14)	0.05711 (17)	0.44985 (7)	0.0152 (3)	
H1A	−0.0739	0.0653	0.4910	0.018*	
C2A	0.02250 (15)	0.17288 (17)	0.43916 (7)	0.0163 (4)	
H2A	−0.0080	0.2577	0.4522	0.020*	
C3A	0.14809 (15)	0.13639 (17)	0.47939 (7)	0.0164 (4)	
H3A	0.2172	0.1681	0.4623	0.020*	
C4A	0.14303 (15)	−0.01529 (17)	0.48322 (7)	0.0170 (4)	
H4A	0.2092	−0.0575	0.4671	0.020*	
C5A	−0.17374 (15)	0.03966 (17)	0.40139 (7)	0.0153 (3)	
H5A	−0.2085	−0.0486	0.4068	0.018*	
C6A	−0.26913 (15)	0.14410 (17)	0.40528 (7)	0.0170 (3)	
H6A1	−0.2334	0.2322	0.4028	0.020*	
H6A2	−0.3408	0.1344	0.3699	0.020*	
C7A	−0.04092 (15)	0.12469 (17)	0.26366 (7)	0.0165 (3)	
C8A	−0.13660 (15)	0.08451 (17)	0.21478 (8)	0.0187 (4)	
H8A	−0.2103	0.0522	0.2230	0.022*	
C9A	−0.12639 (15)	0.09073 (18)	0.15468 (8)	0.0190 (4)	
H9A	−0.1932	0.0626	0.1226	0.023*	
C10A	−0.01962 (15)	0.13757 (17)	0.14030 (8)	0.0168 (3)	
C11A	0.07554 (15)	0.17926 (17)	0.18901 (8)	0.0183 (4)	
H11A	0.1489	0.2123	0.1808	0.022*	
C12A	0.06513 (15)	0.17350 (17)	0.24947 (8)	0.0180 (4)	
H12A	0.1312	0.2032	0.2815	0.022*	
C13A	0.14908 (14)	0.08726 (17)	0.64508 (7)	0.0163 (4)	
C14A	0.14777 (15)	0.21093 (18)	0.67181 (8)	0.0191 (4)	
H14A	0.1614	0.2867	0.6498	0.023*	
C15A	0.12716 (15)	0.22555 (18)	0.72956 (8)	0.0193 (4)	
H15A	0.1273	0.3110	0.7464	0.023*	
C16A	0.10604 (14)	0.11714 (17)	0.76369 (7)	0.0167 (4)	
C17A	0.10985 (15)	−0.00752 (17)	0.73780 (8)	0.0186 (4)	
H17A	0.0977	−0.0834	0.7601	0.022*	
C18A	0.13121 (15)	−0.02131 (17)	0.67968 (8)	0.0188 (4)	
H18A	0.1337	−0.1068	0.6632	0.023*	
C19A	0.07786 (15)	0.13829 (17)	0.82624 (7)	0.0181 (4)	
C20A	0.18873 (16)	0.2038 (2)	0.87000 (8)	0.0242 (4)	
H20A	0.2622	0.1502	0.8724	0.036*	
H20B	0.1731	0.2116	0.9108	0.036*	
H20C	0.2015	0.2913	0.8546	0.036*	
C21A	−0.03485 (16)	0.22779 (19)	0.81846 (8)	0.0232 (4)	
H21A	−0.0169	0.3134	0.8028	0.035*	
H21B	−0.0549	0.2393	0.8581	0.035*	
H21C	−0.1047	0.1877	0.7894	0.035*	
C22A	0.05076 (17)	0.00842 (19)	0.85447 (8)	0.0242 (4)	
H22A	−0.0199	−0.0341	0.8270	0.036*	
H22B	0.0323	0.0254	0.8941	0.036*	
H22C	0.1225	−0.0492	0.8603	0.036*	
C23A	−0.01052 (15)	0.13871 (18)	0.07325 (7)	0.0176 (4)	
C24A	−0.11940 (17)	0.2150 (2)	0.03317 (8)	0.0245 (4)	
H24A	−0.1966	0.1739	0.0362	0.037*	
H24B	−0.1136	0.2134	−0.0096	0.037*	
H24C	−0.1172	0.3060	0.0474	0.037*	
C25A	−0.01485 (17)	−0.00393 (19)	0.04997 (8)	0.0232 (4)	
H25A	−0.0914	−0.0454	0.0537	0.035*	
H25B	0.0552	−0.0528	0.0745	0.035*	
H25C	−0.0111	−0.0042	0.0069	0.035*	
C26A	0.10810 (16)	0.2025 (2)	0.06552 (8)	0.0243 (4)	
H26A	0.1089	0.2025	0.0221	0.036*	
H26B	0.1788	0.1529	0.0889	0.036*	
H26C	0.1126	0.2931	0.0806	0.036*	
O1A	0.02515 (10)	−0.05418 (11)	0.44963 (5)	0.0169 (3)	
O2A	−0.15223 (10)	0.04640 (12)	0.34114 (5)	0.0182 (3)	
O3A	0.03041 (10)	0.17685 (12)	0.37658 (5)	0.0181 (3)	
O4A	0.15721 (10)	−0.04286 (12)	0.54725 (5)	0.0186 (3)	
O5A	0.15740 (10)	0.18162 (12)	0.54097 (5)	0.0181 (3)	
O6A	−0.30884 (10)	0.13444 (13)	0.46061 (5)	0.0205 (3)	
H6A	−0.2547	0.1657	0.4897	0.031*	
B1B	−0.37416 (18)	0.0014 (2)	0.69984 (9)	0.0191 (4)	
B2B	−0.50375 (17)	−0.1162 (2)	0.45330 (9)	0.0172 (4)	
C1B	−0.32186 (15)	−0.02484 (17)	0.58744 (7)	0.0162 (3)	
H1B	−0.3047	0.0028	0.5477	0.019*	
C2B	−0.45126 (15)	0.01735 (17)	0.58916 (7)	0.0162 (3)	
H2B	−0.4706	0.1079	0.5725	0.019*	
C3B	−0.53087 (15)	−0.08554 (17)	0.54920 (7)	0.0169 (4)	
H3B	−0.6026	−0.1116	0.5655	0.020*	
C4B	−0.44075 (15)	−0.20161 (17)	0.54991 (8)	0.0171 (4)	
H4B	−0.4734	−0.2852	0.5630	0.020*	
C5B	−0.22174 (16)	0.02487 (18)	0.63990 (7)	0.0195 (4)	
H5B	−0.1460	−0.0280	0.6417	0.023*	
C6B	−0.19178 (16)	0.16913 (19)	0.63265 (8)	0.0215 (4)	
H6B1	−0.2662	0.2231	0.6300	0.026*	
H6B2	−0.1282	0.1984	0.6689	0.026*	
C7B	−0.40312 (16)	−0.01712 (18)	0.76364 (7)	0.0191 (4)	
C8B	−0.31073 (16)	−0.00618 (19)	0.81758 (8)	0.0229 (4)	
H8B	−0.2294	0.0131	0.8151	0.028*	
C9B	−0.33471 (15)	−0.02280 (19)	0.87460 (8)	0.0214 (4)	
H9B	−0.2702	−0.0118	0.9104	0.026*	
C10B	−0.45185 (15)	−0.05533 (17)	0.88046 (7)	0.0167 (3)	
C11B	−0.54493 (16)	−0.06565 (19)	0.82682 (8)	0.0208 (4)	
H11B	−0.6260	−0.0861	0.8293	0.025*	
C12B	−0.52069 (16)	−0.04637 (19)	0.76972 (8)	0.0224 (4)	
H12B	−0.5859	−0.0533	0.7340	0.027*	
C13B	−0.51855 (15)	−0.10119 (17)	0.38352 (8)	0.0170 (4)	
C14B	−0.60372 (16)	−0.01384 (18)	0.34760 (8)	0.0213 (4)	
H14B	−0.6539	0.0387	0.3665	0.026*	
C15B	−0.61530 (16)	−0.00353 (19)	0.28521 (8)	0.0218 (4)	
H15B	−0.6735	0.0560	0.2620	0.026*	
C16B	−0.54304 (15)	−0.07903 (17)	0.25534 (8)	0.0182 (4)	
C17B	−0.45914 (15)	−0.16602 (18)	0.29079 (8)	0.0193 (4)	
H17B	−0.4094	−0.2190	0.2718	0.023*	
C18B	−0.44731 (15)	−0.17626 (17)	0.35355 (8)	0.0184 (4)	
H18B	−0.3891	−0.2360	0.3766	0.022*	
C19B	−0.55901 (16)	−0.06446 (19)	0.18590 (8)	0.0210 (4)	
C20B	−0.68993 (17)	−0.1066 (2)	0.15334 (8)	0.0287 (4)	
H20D	−0.7015	−0.1994	0.1620	0.043*	
H20E	−0.7025	−0.0941	0.1091	0.043*	
H20F	−0.7492	−0.0532	0.1682	0.043*	
C21B	−0.53829 (19)	0.0793 (2)	0.17082 (9)	0.0302 (4)	
H21D	−0.5970	0.1352	0.1848	0.045*	
H21E	−0.5501	0.0892	0.1265	0.045*	
H21F	−0.4545	0.1054	0.1916	0.045*	
C22B	−0.46919 (18)	−0.1506 (2)	0.16226 (9)	0.0292 (5)	
H22D	−0.3849	−0.1255	0.1826	0.044*	
H22E	−0.4826	−0.1387	0.1179	0.044*	
H22F	−0.4825	−0.2428	0.1711	0.044*	
C23B	−0.47421 (15)	−0.07315 (18)	0.94441 (7)	0.0180 (4)	
C24B	−0.46988 (18)	0.0627 (2)	0.97443 (9)	0.0281 (4)	
H24D	−0.3898	0.1032	0.9767	0.042*	
H24E	−0.4824	0.0533	1.0158	0.042*	
H24F	−0.5347	0.1183	0.9499	0.042*	
C25B	−0.37355 (16)	−0.1596 (2)	0.98413 (8)	0.0228 (4)	
H25D	−0.2933	−0.1184	0.9876	0.034*	
H25E	−0.3746	−0.2462	0.9651	0.034*	
H25F	−0.3886	−0.1693	1.0250	0.034*	
C26B	−0.59886 (16)	−0.1354 (2)	0.94248 (8)	0.0239 (4)	
H26D	−0.6063	−0.1511	0.9844	0.036*	
H26E	−0.6058	−0.2189	0.9203	0.036*	
H26F	−0.6644	−0.0761	0.9215	0.036*	
O1B	−0.32857 (10)	−0.16637 (12)	0.58971 (5)	0.0175 (3)	
O2B	−0.25412 (11)	0.01234 (13)	0.69747 (5)	0.0232 (3)	
O3B	−0.47056 (10)	0.00418 (12)	0.64942 (5)	0.0193 (3)	
O4B	−0.42916 (10)	−0.20952 (12)	0.48781 (5)	0.0182 (3)	
O5B	−0.56770 (10)	−0.04289 (12)	0.48642 (5)	0.0175 (3)	
O6B	−0.14817 (11)	0.18677 (13)	0.57813 (5)	0.0233 (3)	
H6B	−0.0839	0.2323	0.5864	0.035*	

### Atomic displacement parameters (Å^2^)

**Table:**

	*U*^11^	*U*^22^	*U*^33^	*U*^12^	*U*^13^	*U*^23^
B1A	0.0159 (9)	0.0133 (10)	0.0197 (10)	0.0036 (8)	0.0051 (8)	0.0009 (8)
B2A	0.0104 (8)	0.0175 (10)	0.0217 (10)	0.0003 (8)	0.0032 (7)	0.0000 (8)
C1A	0.0170 (8)	0.0136 (8)	0.0164 (8)	0.0015 (7)	0.0069 (7)	−0.0006 (7)
C2A	0.0196 (8)	0.0151 (9)	0.0150 (8)	−0.0001 (7)	0.0058 (7)	−0.0007 (7)
C3A	0.0151 (8)	0.0194 (9)	0.0153 (8)	−0.0024 (7)	0.0048 (7)	−0.0010 (7)
C4A	0.0149 (8)	0.0192 (9)	0.0170 (8)	0.0004 (7)	0.0044 (7)	−0.0009 (7)
C5A	0.0162 (8)	0.0172 (9)	0.0137 (8)	−0.0014 (7)	0.0062 (6)	0.0002 (7)
C6A	0.0168 (8)	0.0179 (9)	0.0170 (8)	0.0002 (7)	0.0052 (7)	0.0019 (7)
C7A	0.0194 (8)	0.0139 (8)	0.0167 (8)	0.0030 (7)	0.0055 (7)	−0.0001 (7)
C8A	0.0170 (8)	0.0175 (9)	0.0231 (9)	−0.0009 (7)	0.0078 (7)	0.0022 (7)
C9A	0.0173 (8)	0.0193 (9)	0.0186 (9)	−0.0015 (7)	0.0013 (7)	−0.0008 (7)
C10A	0.0193 (8)	0.0129 (8)	0.0186 (9)	0.0024 (7)	0.0058 (7)	0.0012 (7)
C11A	0.0160 (8)	0.0176 (9)	0.0220 (9)	0.0002 (7)	0.0060 (7)	0.0019 (7)
C12A	0.0176 (8)	0.0168 (9)	0.0185 (9)	0.0001 (7)	0.0021 (7)	−0.0011 (7)
C13A	0.0112 (8)	0.0193 (9)	0.0176 (8)	0.0003 (7)	0.0016 (6)	0.0015 (7)
C14A	0.0185 (9)	0.0175 (9)	0.0214 (9)	−0.0023 (7)	0.0052 (7)	0.0030 (7)
C15A	0.0203 (9)	0.0146 (9)	0.0229 (9)	−0.0028 (7)	0.0051 (7)	−0.0026 (7)
C16A	0.0125 (8)	0.0198 (9)	0.0175 (9)	−0.0012 (7)	0.0030 (7)	−0.0005 (7)
C17A	0.0207 (9)	0.0147 (9)	0.0203 (9)	−0.0007 (7)	0.0048 (7)	0.0031 (7)
C18A	0.0200 (9)	0.0150 (9)	0.0209 (9)	0.0011 (7)	0.0038 (7)	−0.0018 (7)
C19A	0.0190 (8)	0.0179 (9)	0.0185 (9)	−0.0034 (7)	0.0067 (7)	−0.0007 (7)
C20A	0.0251 (9)	0.0287 (11)	0.0189 (9)	−0.0051 (8)	0.0054 (7)	−0.0022 (8)
C21A	0.0225 (9)	0.0248 (10)	0.0238 (10)	0.0000 (8)	0.0086 (8)	−0.0025 (8)
C22A	0.0287 (10)	0.0258 (10)	0.0206 (9)	−0.0026 (8)	0.0106 (8)	0.0019 (8)
C23A	0.0201 (8)	0.0191 (9)	0.0138 (8)	−0.0005 (7)	0.0045 (7)	−0.0003 (7)
C24A	0.0273 (10)	0.0274 (10)	0.0190 (9)	0.0037 (8)	0.0057 (8)	0.0028 (8)
C25A	0.0295 (10)	0.0230 (10)	0.0181 (9)	−0.0008 (8)	0.0079 (8)	−0.0024 (7)
C26A	0.0267 (10)	0.0296 (11)	0.0185 (9)	−0.0036 (8)	0.0091 (7)	0.0032 (8)
O1A	0.0167 (6)	0.0136 (6)	0.0208 (6)	0.0008 (5)	0.0053 (5)	−0.0020 (5)
O2A	0.0180 (6)	0.0221 (7)	0.0150 (6)	−0.0032 (5)	0.0052 (5)	−0.0024 (5)
O3A	0.0206 (6)	0.0191 (6)	0.0151 (6)	−0.0036 (5)	0.0054 (5)	0.0015 (5)
O4A	0.0199 (6)	0.0183 (6)	0.0175 (6)	0.0021 (5)	0.0041 (5)	0.0015 (5)
O5A	0.0194 (6)	0.0180 (6)	0.0168 (6)	−0.0032 (5)	0.0041 (5)	−0.0017 (5)
O6A	0.0182 (6)	0.0268 (7)	0.0178 (6)	0.0000 (5)	0.0069 (5)	0.0002 (5)
B1B	0.0200 (10)	0.0185 (10)	0.0183 (10)	−0.0009 (8)	0.0038 (8)	−0.0008 (8)
B2B	0.0138 (9)	0.0154 (10)	0.0219 (10)	−0.0043 (8)	0.0033 (8)	−0.0025 (8)
C1B	0.0160 (8)	0.0172 (9)	0.0163 (8)	−0.0001 (7)	0.0058 (7)	0.0016 (7)
C2B	0.0183 (8)	0.0165 (9)	0.0142 (8)	0.0010 (7)	0.0048 (7)	0.0012 (7)
C3B	0.0146 (8)	0.0187 (9)	0.0175 (8)	−0.0003 (7)	0.0043 (7)	0.0011 (7)
C4B	0.0161 (8)	0.0164 (9)	0.0189 (9)	−0.0012 (7)	0.0048 (7)	−0.0003 (7)
C5B	0.0183 (9)	0.0257 (10)	0.0152 (8)	−0.0014 (7)	0.0051 (7)	0.0007 (7)
C6B	0.0210 (9)	0.0255 (10)	0.0178 (9)	−0.0038 (8)	0.0046 (7)	−0.0030 (8)
C7B	0.0209 (9)	0.0188 (9)	0.0174 (9)	0.0006 (7)	0.0042 (7)	0.0015 (7)
C8B	0.0176 (9)	0.0287 (10)	0.0230 (9)	−0.0034 (8)	0.0057 (7)	0.0039 (8)
C9B	0.0178 (9)	0.0276 (10)	0.0162 (9)	−0.0016 (8)	−0.0007 (7)	0.0015 (7)
C10B	0.0202 (8)	0.0131 (8)	0.0173 (8)	0.0015 (7)	0.0054 (7)	0.0001 (7)
C11B	0.0162 (8)	0.0268 (11)	0.0200 (9)	−0.0018 (8)	0.0056 (7)	0.0001 (7)
C12B	0.0194 (9)	0.0281 (10)	0.0174 (9)	−0.0007 (8)	−0.0001 (7)	−0.0002 (8)
C13B	0.0152 (8)	0.0164 (9)	0.0192 (9)	−0.0030 (7)	0.0034 (7)	−0.0023 (7)
C14B	0.0205 (9)	0.0208 (10)	0.0232 (9)	0.0019 (8)	0.0066 (7)	−0.0043 (7)
C15B	0.0185 (9)	0.0218 (10)	0.0234 (9)	0.0065 (8)	0.0018 (7)	0.0009 (8)
C16B	0.0178 (8)	0.0182 (9)	0.0191 (9)	−0.0009 (7)	0.0054 (7)	−0.0003 (7)
C17B	0.0178 (9)	0.0206 (9)	0.0199 (9)	0.0016 (7)	0.0056 (7)	−0.0026 (7)
C18B	0.0147 (8)	0.0164 (9)	0.0233 (9)	0.0007 (7)	0.0031 (7)	0.0015 (7)
C19B	0.0216 (9)	0.0251 (10)	0.0168 (9)	0.0031 (8)	0.0057 (7)	0.0007 (7)
C20B	0.0269 (10)	0.0390 (12)	0.0186 (10)	0.0013 (9)	0.0024 (8)	−0.0013 (9)
C21B	0.0335 (11)	0.0312 (11)	0.0263 (10)	−0.0010 (9)	0.0082 (8)	0.0059 (9)
C22B	0.0312 (11)	0.0383 (12)	0.0200 (10)	0.0076 (9)	0.0098 (8)	0.0002 (9)
C23B	0.0188 (8)	0.0203 (9)	0.0152 (8)	0.0004 (7)	0.0051 (7)	0.0020 (7)
C24B	0.0347 (11)	0.0248 (10)	0.0266 (10)	0.0016 (9)	0.0110 (8)	−0.0043 (8)
C25B	0.0192 (9)	0.0303 (11)	0.0199 (9)	0.0001 (8)	0.0066 (7)	0.0053 (8)
C26B	0.0197 (9)	0.0307 (11)	0.0227 (10)	−0.0004 (8)	0.0076 (8)	0.0050 (8)
O1B	0.0158 (6)	0.0157 (6)	0.0201 (6)	0.0010 (5)	0.0026 (5)	0.0009 (5)
O2B	0.0206 (6)	0.0344 (8)	0.0143 (6)	−0.0040 (6)	0.0039 (5)	0.0018 (5)
O3B	0.0184 (6)	0.0241 (7)	0.0159 (6)	0.0038 (5)	0.0053 (5)	0.0005 (5)
O4B	0.0202 (6)	0.0170 (6)	0.0172 (6)	0.0022 (5)	0.0044 (5)	−0.0017 (5)
O5B	0.0166 (6)	0.0205 (6)	0.0147 (6)	0.0016 (5)	0.0021 (5)	0.0003 (5)
O6B	0.0229 (7)	0.0261 (7)	0.0214 (7)	−0.0094 (6)	0.0062 (5)	0.0011 (5)

### Geometric parameters (Å, °)

**Table:**

B1A—O2A	1.366 (2)	B1B—O3B	1.365 (2)
B1A—O3A	1.368 (2)	B1B—O2B	1.367 (2)
B1A—C7A	1.569 (2)	B1B—C7B	1.559 (2)
B2A—O5A	1.369 (2)	B2B—O5B	1.375 (2)
B2A—O4A	1.376 (2)	B2B—O4B	1.376 (2)
B2A—C13A	1.550 (2)	B2B—C13B	1.546 (2)
C1A—O1A	1.454 (2)	C1B—O1B	1.446 (2)
C1A—C5A	1.509 (2)	C1B—C5B	1.506 (2)
C1A—C2A	1.525 (2)	C1B—C2B	1.526 (2)
C1A—H1A	1.0000	C1B—H1B	1.0000
C2A—O3A	1.4334 (18)	C2B—O3B	1.4310 (18)
C2A—C3A	1.526 (2)	C2B—C3B	1.522 (2)
C2A—H2A	1.0000	C2B—H2B	1.0000
C3A—O5A	1.4400 (19)	C3B—O5B	1.4392 (19)
C3A—C4A	1.550 (2)	C3B—C4B	1.555 (2)
C3A—H3A	1.0000	C3B—H3B	1.0000
C4A—O1A	1.4125 (19)	C4B—O1B	1.404 (2)
C4A—O4A	1.4386 (19)	C4B—O4B	1.4373 (19)
C4A—H4A	1.0000	C4B—H4B	1.0000
C5A—O2A	1.4362 (18)	C5B—O2B	1.4344 (19)
C5A—C6A	1.528 (2)	C5B—C6B	1.526 (3)
C5A—H5A	1.0000	C5B—H5B	1.0000
C6A—O6A	1.4248 (18)	C6B—O6B	1.4394 (19)
C6A—H6A1	0.9900	C6B—H6B1	0.9900
C6A—H6A2	0.9900	C6B—H6B2	0.9900
C7A—C12A	1.398 (2)	C7B—C12B	1.393 (2)
C7A—C8A	1.399 (2)	C7B—C8B	1.397 (2)
C8A—C9A	1.387 (2)	C8B—C9B	1.385 (2)
C8A—H8A	0.9500	C8B—H8B	0.9500
C9A—C10A	1.401 (2)	C9B—C10B	1.394 (2)
C9A—H9A	0.9500	C9B—H9B	0.9500
C10A—C11A	1.398 (2)	C10B—C11B	1.395 (2)
C10A—C23A	1.537 (2)	C10B—C23B	1.531 (2)
C11A—C12A	1.395 (2)	C11B—C12B	1.391 (2)
C11A—H11A	0.9500	C11B—H11B	0.9500
C12A—H12A	0.9500	C12B—H12B	0.9500
C13A—C18A	1.395 (2)	C13B—C18B	1.394 (2)
C13A—C14A	1.398 (2)	C13B—C14B	1.409 (2)
C14A—C15A	1.383 (2)	C14B—C15B	1.382 (2)
C14A—H14A	0.9500	C14B—H14B	0.9500
C15A—C16A	1.399 (2)	C15B—C16B	1.403 (2)
C15A—H15A	0.9500	C15B—H15B	0.9500
C16A—C17A	1.403 (2)	C16B—C17B	1.395 (2)
C16A—C19A	1.532 (2)	C16B—C19B	1.536 (2)
C17A—C18A	1.393 (2)	C17B—C18B	1.390 (2)
C17A—H17A	0.9500	C17B—H17B	0.9500
C18A—H18A	0.9500	C18B—H18B	0.9500
C19A—C22A	1.530 (2)	C19B—C22B	1.528 (3)
C19A—C21A	1.536 (2)	C19B—C21B	1.534 (3)
C19A—C20A	1.539 (2)	C19B—C20B	1.536 (3)
C20A—H20A	0.9800	C20B—H20D	0.9800
C20A—H20B	0.9800	C20B—H20E	0.9800
C20A—H20C	0.9800	C20B—H20F	0.9800
C21A—H21A	0.9800	C21B—H21D	0.9800
C21A—H21B	0.9800	C21B—H21E	0.9800
C21A—H21C	0.9800	C21B—H21F	0.9800
C22A—H22A	0.9800	C22B—H22D	0.9800
C22A—H22B	0.9800	C22B—H22E	0.9800
C22A—H22C	0.9800	C22B—H22F	0.9800
C23A—C26A	1.531 (2)	C23B—C26B	1.529 (2)
C23A—C24A	1.542 (2)	C23B—C24B	1.536 (3)
C23A—C25A	1.542 (3)	C23B—C25B	1.537 (2)
C24A—H24A	0.9800	C24B—H24D	0.9800
C24A—H24B	0.9800	C24B—H24E	0.9800
C24A—H24C	0.9800	C24B—H24F	0.9800
C25A—H25A	0.9800	C25B—H25D	0.9800
C25A—H25B	0.9800	C25B—H25E	0.9800
C25A—H25C	0.9800	C25B—H25F	0.9800
C26A—H26A	0.9800	C26B—H26D	0.9800
C26A—H26B	0.9800	C26B—H26E	0.9800
C26A—H26C	0.9800	C26B—H26F	0.9800
O6A—H6A	0.8400	O6B—H6B	0.8400
			
O2A—B1A—O3A	123.84 (15)	O3B—B1B—O2B	123.81 (16)
O2A—B1A—C7A	117.46 (15)	O3B—B1B—C7B	117.83 (15)
O3A—B1A—C7A	118.70 (15)	O2B—B1B—C7B	118.36 (15)
O5A—B2A—O4A	113.24 (14)	O5B—B2B—O4B	113.35 (15)
O5A—B2A—C13A	122.11 (16)	O5B—B2B—C13B	123.78 (16)
O4A—B2A—C13A	124.50 (16)	O4B—B2B—C13B	122.80 (16)
O1A—C1A—C5A	110.22 (13)	O1B—C1B—C5B	110.08 (14)
O1A—C1A—C2A	102.63 (12)	O1B—C1B—C2B	102.82 (13)
C5A—C1A—C2A	114.59 (14)	C5B—C1B—C2B	114.82 (14)
O1A—C1A—H1A	109.7	O1B—C1B—H1B	109.6
C5A—C1A—H1A	109.7	C5B—C1B—H1B	109.6
C2A—C1A—H1A	109.7	C2B—C1B—H1B	109.6
O3A—C2A—C1A	110.67 (13)	O3B—C2B—C3B	107.02 (13)
O3A—C2A—C3A	108.68 (12)	O3B—C2B—C1B	111.29 (13)
C1A—C2A—C3A	101.71 (13)	C3B—C2B—C1B	102.56 (13)
O3A—C2A—H2A	111.8	O3B—C2B—H2B	111.8
C1A—C2A—H2A	111.8	C3B—C2B—H2B	111.8
C3A—C2A—H2A	111.8	C1B—C2B—H2B	111.8
O5A—C3A—C2A	109.46 (13)	O5B—C3B—C2B	111.05 (14)
O5A—C3A—C4A	105.12 (13)	O5B—C3B—C4B	105.70 (13)
C2A—C3A—C4A	103.59 (14)	C2B—C3B—C4B	103.33 (13)
O5A—C3A—H3A	112.7	O5B—C3B—H3B	112.1
C2A—C3A—H3A	112.7	C2B—C3B—H3B	112.1
C4A—C3A—H3A	112.7	C4B—C3B—H3B	112.1
O1A—C4A—O4A	110.00 (12)	O1B—C4B—O4B	110.79 (13)
O1A—C4A—C3A	107.00 (13)	O1B—C4B—C3B	107.05 (13)
O4A—C4A—C3A	104.70 (13)	O4B—C4B—C3B	104.23 (13)
O1A—C4A—H4A	111.6	O1B—C4B—H4B	111.5
O4A—C4A—H4A	111.6	O4B—C4B—H4B	111.5
C3A—C4A—H4A	111.6	C3B—C4B—H4B	111.5
O2A—C5A—C1A	110.87 (12)	O2B—C5B—C1B	111.88 (13)
O2A—C5A—C6A	107.62 (12)	O2B—C5B—C6B	107.07 (14)
C1A—C5A—C6A	112.46 (14)	C1B—C5B—C6B	112.48 (15)
O2A—C5A—H5A	108.6	O2B—C5B—H5B	108.4
C1A—C5A—H5A	108.6	C1B—C5B—H5B	108.4
C6A—C5A—H5A	108.6	C6B—C5B—H5B	108.4
O6A—C6A—C5A	112.05 (13)	O6B—C6B—C5B	109.96 (14)
O6A—C6A—H6A1	109.2	O6B—C6B—H6B1	109.7
C5A—C6A—H6A1	109.2	C5B—C6B—H6B1	109.7
O6A—C6A—H6A2	109.2	O6B—C6B—H6B2	109.7
C5A—C6A—H6A2	109.2	C5B—C6B—H6B2	109.7
H6A1—C6A—H6A2	107.9	H6B1—C6B—H6B2	108.2
C12A—C7A—C8A	117.31 (15)	C12B—C7B—C8B	116.97 (15)
C12A—C7A—B1A	122.22 (15)	C12B—C7B—B1B	122.14 (15)
C8A—C7A—B1A	120.47 (15)	C8B—C7B—B1B	120.88 (15)
C9A—C8A—C7A	121.56 (15)	C9B—C8B—C7B	121.65 (16)
C9A—C8A—H8A	119.2	C9B—C8B—H8B	119.2
C7A—C8A—H8A	119.2	C7B—C8B—H8B	119.2
C8A—C9A—C10A	121.35 (15)	C8B—C9B—C10B	121.21 (15)
C8A—C9A—H9A	119.3	C8B—C9B—H9B	119.4
C10A—C9A—H9A	119.3	C10B—C9B—H9B	119.4
C11A—C10A—C9A	117.20 (15)	C9B—C10B—C11B	117.51 (15)
C11A—C10A—C23A	123.11 (14)	C9B—C10B—C23B	119.47 (14)
C9A—C10A—C23A	119.68 (14)	C11B—C10B—C23B	122.97 (15)
C12A—C11A—C10A	121.47 (15)	C12B—C11B—C10B	120.96 (16)
C12A—C11A—H11A	119.3	C12B—C11B—H11B	119.5
C10A—C11A—H11A	119.3	C10B—C11B—H11B	119.5
C11A—C12A—C7A	121.10 (15)	C11B—C12B—C7B	121.66 (15)
C11A—C12A—H12A	119.4	C11B—C12B—H12B	119.2
C7A—C12A—H12A	119.4	C7B—C12B—H12B	119.2
C18A—C13A—C14A	117.17 (15)	C18B—C13B—C14B	117.31 (15)
C18A—C13A—B2A	122.09 (15)	C18B—C13B—B2B	120.32 (15)
C14A—C13A—B2A	120.48 (15)	C14B—C13B—B2B	122.36 (15)
C15A—C14A—C13A	121.55 (16)	C15B—C14B—C13B	120.90 (16)
C15A—C14A—H14A	119.2	C15B—C14B—H14B	119.6
C13A—C14A—H14A	119.2	C13B—C14B—H14B	119.6
C14A—C15A—C16A	121.40 (16)	C14B—C15B—C16B	121.53 (16)
C14A—C15A—H15A	119.3	C14B—C15B—H15B	119.2
C16A—C15A—H15A	119.3	C16B—C15B—H15B	119.2
C15A—C16A—C17A	117.44 (15)	C17B—C16B—C15B	117.62 (16)
C15A—C16A—C19A	119.64 (15)	C17B—C16B—C19B	122.60 (15)
C17A—C16A—C19A	122.91 (15)	C15B—C16B—C19B	119.78 (15)
C18A—C17A—C16A	120.74 (15)	C18B—C17B—C16B	120.88 (16)
C18A—C17A—H17A	119.6	C18B—C17B—H17B	119.6
C16A—C17A—H17A	119.6	C16B—C17B—H17B	119.6
C17A—C18A—C13A	121.67 (16)	C17B—C18B—C13B	121.76 (16)
C17A—C18A—H18A	119.2	C17B—C18B—H18B	119.1
C13A—C18A—H18A	119.2	C13B—C18B—H18B	119.1
C22A—C19A—C16A	111.50 (14)	C22B—C19B—C21B	108.83 (15)
C22A—C19A—C21A	108.60 (14)	C22B—C19B—C16B	112.05 (15)
C16A—C19A—C21A	109.16 (14)	C21B—C19B—C16B	109.33 (15)
C22A—C19A—C20A	108.73 (14)	C22B—C19B—C20B	108.07 (15)
C16A—C19A—C20A	109.43 (13)	C21B—C19B—C20B	109.92 (16)
C21A—C19A—C20A	109.38 (15)	C16B—C19B—C20B	108.62 (14)
C19A—C20A—H20A	109.5	C19B—C20B—H20D	109.5
C19A—C20A—H20B	109.5	C19B—C20B—H20E	109.5
H20A—C20A—H20B	109.5	H20D—C20B—H20E	109.5
C19A—C20A—H20C	109.5	C19B—C20B—H20F	109.5
H20A—C20A—H20C	109.5	H20D—C20B—H20F	109.5
H20B—C20A—H20C	109.5	H20E—C20B—H20F	109.5
C19A—C21A—H21A	109.5	C19B—C21B—H21D	109.5
C19A—C21A—H21B	109.5	C19B—C21B—H21E	109.5
H21A—C21A—H21B	109.5	H21D—C21B—H21E	109.5
C19A—C21A—H21C	109.5	C19B—C21B—H21F	109.5
H21A—C21A—H21C	109.5	H21D—C21B—H21F	109.5
H21B—C21A—H21C	109.5	H21E—C21B—H21F	109.5
C19A—C22A—H22A	109.5	C19B—C22B—H22D	109.5
C19A—C22A—H22B	109.5	C19B—C22B—H22E	109.5
H22A—C22A—H22B	109.5	H22D—C22B—H22E	109.5
C19A—C22A—H22C	109.5	C19B—C22B—H22F	109.5
H22A—C22A—H22C	109.5	H22D—C22B—H22F	109.5
H22B—C22A—H22C	109.5	H22E—C22B—H22F	109.5
C26A—C23A—C10A	112.52 (13)	C26B—C23B—C10B	112.44 (14)
C26A—C23A—C24A	107.95 (15)	C26B—C23B—C24B	108.49 (15)
C10A—C23A—C24A	110.19 (14)	C10B—C23B—C24B	108.16 (14)
C26A—C23A—C25A	108.55 (15)	C26B—C23B—C25B	108.54 (15)
C10A—C23A—C25A	108.81 (14)	C10B—C23B—C25B	110.41 (13)
C24A—C23A—C25A	108.75 (14)	C24B—C23B—C25B	108.72 (14)
C23A—C24A—H24A	109.5	C23B—C24B—H24D	109.5
C23A—C24A—H24B	109.5	C23B—C24B—H24E	109.5
H24A—C24A—H24B	109.5	H24D—C24B—H24E	109.5
C23A—C24A—H24C	109.5	C23B—C24B—H24F	109.5
H24A—C24A—H24C	109.5	H24D—C24B—H24F	109.5
H24B—C24A—H24C	109.5	H24E—C24B—H24F	109.5
C23A—C25A—H25A	109.5	C23B—C25B—H25D	109.5
C23A—C25A—H25B	109.5	C23B—C25B—H25E	109.5
H25A—C25A—H25B	109.5	H25D—C25B—H25E	109.5
C23A—C25A—H25C	109.5	C23B—C25B—H25F	109.5
H25A—C25A—H25C	109.5	H25D—C25B—H25F	109.5
H25B—C25A—H25C	109.5	H25E—C25B—H25F	109.5
C23A—C26A—H26A	109.5	C23B—C26B—H26D	109.5
C23A—C26A—H26B	109.5	C23B—C26B—H26E	109.5
H26A—C26A—H26B	109.5	H26D—C26B—H26E	109.5
C23A—C26A—H26C	109.5	C23B—C26B—H26F	109.5
H26A—C26A—H26C	109.5	H26D—C26B—H26F	109.5
H26B—C26A—H26C	109.5	H26E—C26B—H26F	109.5
C4A—O1A—C1A	106.52 (12)	C4B—O1B—C1B	106.21 (13)
B1A—O2A—C5A	120.33 (13)	B1B—O2B—C5B	120.82 (13)
B1A—O3A—C2A	121.39 (13)	B1B—O3B—C2B	121.14 (13)
B2A—O4A—C4A	108.30 (13)	B2B—O4B—C4B	108.85 (13)
B2A—O5A—C3A	108.27 (13)	B2B—O5B—C3B	107.75 (13)
C6A—O6A—H6A	109.5	C6B—O6B—H6B	109.5
			
O1A—C1A—C2A—O3A	74.30 (15)	O1B—C1B—C2B—O3B	75.41 (16)
C5A—C1A—C2A—O3A	−45.18 (18)	C5B—C1B—C2B—O3B	−44.13 (19)
O1A—C1A—C2A—C3A	−41.03 (15)	O1B—C1B—C2B—C3B	−38.70 (15)
C5A—C1A—C2A—C3A	−160.51 (13)	C5B—C1B—C2B—C3B	−158.24 (14)
O3A—C2A—C3A—O5A	157.08 (13)	O3B—C2B—C3B—O5B	150.91 (12)
C1A—C2A—C3A—O5A	−86.13 (16)	C1B—C2B—C3B—O5B	−91.89 (15)
O3A—C2A—C3A—C4A	−91.21 (15)	O3B—C2B—C3B—C4B	−96.18 (14)
C1A—C2A—C3A—C4A	25.58 (16)	C1B—C2B—C3B—C4B	21.02 (16)
O5A—C3A—C4A—O1A	113.46 (13)	O5B—C3B—C4B—O1B	120.61 (14)
C2A—C3A—C4A—O1A	−1.40 (17)	C2B—C3B—C4B—O1B	3.86 (17)
O5A—C3A—C4A—O4A	−3.28 (16)	O5B—C3B—C4B—O4B	3.17 (16)
C2A—C3A—C4A—O4A	−118.13 (13)	C2B—C3B—C4B—O4B	−113.58 (13)
O1A—C1A—C5A—O2A	−67.31 (16)	O1B—C1B—C5B—O2B	−71.57 (18)
C2A—C1A—C5A—O2A	47.83 (19)	C2B—C1B—C5B—O2B	43.9 (2)
O1A—C1A—C5A—C6A	172.15 (13)	O1B—C1B—C5B—C6B	167.87 (13)
C2A—C1A—C5A—C6A	−72.71 (17)	C2B—C1B—C5B—C6B	−76.71 (18)
O2A—C5A—C6A—O6A	172.42 (12)	O2B—C5B—C6B—O6B	175.05 (12)
C1A—C5A—C6A—O6A	−65.18 (17)	C1B—C5B—C6B—O6B	−61.65 (18)
O2A—B1A—C7A—C12A	−169.26 (16)	O3B—B1B—C7B—C12B	8.0 (3)
O3A—B1A—C7A—C12A	11.4 (2)	O2B—B1B—C7B—C12B	−171.35 (18)
O2A—B1A—C7A—C8A	11.1 (2)	O3B—B1B—C7B—C8B	−172.72 (17)
O3A—B1A—C7A—C8A	−168.20 (16)	O2B—B1B—C7B—C8B	8.0 (3)
C12A—C7A—C8A—C9A	1.0 (3)	C12B—C7B—C8B—C9B	−0.4 (3)
B1A—C7A—C8A—C9A	−179.39 (15)	B1B—C7B—C8B—C9B	−179.74 (18)
C7A—C8A—C9A—C10A	0.0 (3)	C7B—C8B—C9B—C10B	2.0 (3)
C8A—C9A—C10A—C11A	−0.8 (3)	C8B—C9B—C10B—C11B	−2.3 (3)
C8A—C9A—C10A—C23A	178.05 (16)	C8B—C9B—C10B—C23B	179.83 (17)
C9A—C10A—C11A—C12A	0.5 (3)	C9B—C10B—C11B—C12B	1.1 (3)
C23A—C10A—C11A—C12A	−178.26 (16)	C23B—C10B—C11B—C12B	178.85 (17)
C10A—C11A—C12A—C7A	0.5 (3)	C10B—C11B—C12B—C7B	0.5 (3)
C8A—C7A—C12A—C11A	−1.2 (3)	C8B—C7B—C12B—C11B	−0.9 (3)
B1A—C7A—C12A—C11A	179.16 (15)	B1B—C7B—C12B—C11B	178.49 (18)
O5A—B2A—C13A—C18A	170.46 (16)	O5B—B2B—C13B—C18B	177.87 (16)
O4A—B2A—C13A—C18A	−4.8 (2)	O4B—B2B—C13B—C18B	−5.5 (2)
O5A—B2A—C13A—C14A	−3.5 (2)	O5B—B2B—C13B—C14B	−2.8 (3)
O4A—B2A—C13A—C14A	−178.82 (16)	O4B—B2B—C13B—C14B	173.86 (16)
C18A—C13A—C14A—C15A	−1.3 (2)	C18B—C13B—C14B—C15B	−0.1 (3)
B2A—C13A—C14A—C15A	172.97 (14)	B2B—C13B—C14B—C15B	−179.50 (16)
C13A—C14A—C15A—C16A	−0.3 (3)	C13B—C14B—C15B—C16B	−0.1 (3)
C14A—C15A—C16A—C17A	1.7 (2)	C14B—C15B—C16B—C17B	0.4 (3)
C14A—C15A—C16A—C19A	−177.24 (15)	C14B—C15B—C16B—C19B	179.98 (16)
C15A—C16A—C17A—C18A	−1.4 (2)	C15B—C16B—C17B—C18B	−0.5 (2)
C19A—C16A—C17A—C18A	177.48 (15)	C19B—C16B—C17B—C18B	179.93 (16)
C16A—C17A—C18A—C13A	−0.2 (2)	C16B—C17B—C18B—C13B	0.3 (3)
C14A—C13A—C18A—C17A	1.6 (2)	C14B—C13B—C18B—C17B	0.0 (2)
B2A—C13A—C18A—C17A	−172.60 (15)	B2B—C13B—C18B—C17B	179.41 (16)
C15A—C16A—C19A—C22A	176.87 (15)	C17B—C16B—C19B—C22B	−2.6 (2)
C17A—C16A—C19A—C22A	−2.0 (2)	C15B—C16B—C19B—C22B	177.82 (16)
C15A—C16A—C19A—C21A	56.9 (2)	C17B—C16B—C19B—C21B	−123.37 (18)
C17A—C16A—C19A—C21A	−121.98 (17)	C15B—C16B—C19B—C21B	57.1 (2)
C15A—C16A—C19A—C20A	−62.8 (2)	C17B—C16B—C19B—C20B	116.68 (19)
C17A—C16A—C19A—C20A	118.34 (18)	C15B—C16B—C19B—C20B	−62.9 (2)
C11A—C10A—C23A—C26A	−5.6 (2)	C9B—C10B—C23B—C26B	−168.22 (16)
C9A—C10A—C23A—C26A	175.65 (16)	C11B—C10B—C23B—C26B	14.1 (2)
C11A—C10A—C23A—C24A	−126.13 (18)	C9B—C10B—C23B—C24B	72.0 (2)
C9A—C10A—C23A—C24A	55.1 (2)	C11B—C10B—C23B—C24B	−105.69 (19)
C11A—C10A—C23A—C25A	114.73 (18)	C9B—C10B—C23B—C25B	−46.8 (2)
C9A—C10A—C23A—C25A	−64.0 (2)	C11B—C10B—C23B—C25B	135.47 (18)
O4A—C4A—O1A—C1A	87.99 (15)	O4B—C4B—O1B—C1B	83.90 (15)
C3A—C4A—O1A—C1A	−25.19 (16)	C3B—C4B—O1B—C1B	−29.15 (16)
C5A—C1A—O1A—C4A	164.40 (13)	C5B—C1B—O1B—C4B	165.60 (12)
C2A—C1A—O1A—C4A	41.93 (15)	C2B—C1B—O1B—C4B	42.82 (15)
O3A—B1A—O2A—C5A	2.6 (2)	O3B—B1B—O2B—C5B	−1.5 (3)
C7A—B1A—O2A—C5A	−176.74 (14)	C7B—B1B—O2B—C5B	177.80 (15)
C1A—C5A—O2A—B1A	−26.3 (2)	C1B—C5B—O2B—B1B	−21.1 (2)
C6A—C5A—O2A—B1A	97.08 (16)	C6B—C5B—O2B—B1B	102.53 (18)
O2A—B1A—O3A—C2A	0.2 (2)	O2B—B1B—O3B—C2B	0.9 (3)
C7A—B1A—O3A—C2A	179.45 (14)	C7B—B1B—O3B—C2B	−178.35 (15)
C1A—C2A—O3A—B1A	21.1 (2)	C3B—C2B—O3B—B1B	133.04 (16)
C3A—C2A—O3A—B1A	131.94 (16)	C1B—C2B—O3B—B1B	21.7 (2)
O5A—B2A—O4A—C4A	−6.34 (18)	O5B—B2B—O4B—C4B	−0.18 (19)
C13A—B2A—O4A—C4A	169.33 (15)	C13B—B2B—O4B—C4B	−177.13 (15)
O1A—C4A—O4A—B2A	−109.01 (15)	O1B—C4B—O4B—B2B	−116.70 (15)
C3A—C4A—O4A—B2A	5.65 (16)	C3B—C4B—O4B—B2B	−1.87 (16)
O4A—B2A—O5A—C3A	4.07 (18)	O4B—B2B—O5B—C3B	2.35 (19)
C13A—B2A—O5A—C3A	−171.72 (14)	C13B—B2B—O5B—C3B	179.26 (15)
C2A—C3A—O5A—B2A	110.44 (15)	C2B—C3B—O5B—B2B	108.06 (15)
C4A—C3A—O5A—B2A	−0.26 (17)	C4B—C3B—O5B—B2B	−3.34 (17)

### Hydrogen-bond geometry (Å, °)

**Table:**

*D*—H···*A*	*D*—H	H···*A*	*D*···*A*	*D*—H···*A*
O6A—H6A···O6B	0.84	2.07	2.8660 (17)	158
O6B—H6B···O1A^i^	0.84	2.47	3.1131 (17)	134
C1B—H1B···O6A	1.00	2.37	3.3180 (21)	159
C2B—H2B···O4B^ii^	1.00	2.41	3.3794 (20)	162
C4A—H4A···O5B^iii^	1.00	2.44	3.2460 (21)	137
C4B—H4B···O6A^iv^	1.00	2.51	3.2313 (21)	129
C3A—H3A···O1B^i^	1.00	2.55	3.4799 (22)	155
C6A—H6A1···O4A^i^	0.99	2.60	3.4994 (21)	150
C1A—H1A···O6B	1.00	2.62	3.5533 (21)	155
C2A—H2A···O4A^i^	1.00	2.64	3.5867 (21)	159
C14A—H14A···O2A^i^	0.95	2.66	3.4326 (22)	139
C3A—H3A···O4B^i^	1.00	2.68	3.4424 (20)	133
C3B—H3B···O4A^v^	1.00	2.72	3.5216 (21)	137

Symmetry codes: (i) −*x*, *y*+1/2, −*z*+1; (ii) −*x*−1, *y*+1/2, −*z*+1; (iii) *x*+1, *y*, *z*; (iv) −*x*−1, *y*−1/2, −*z*+1; (v) *x*−1, *y*, *z*.
